# Comparison of the Interactions of Different Growth Factors and Glycosaminoglycans

**DOI:** 10.3390/molecules24183360

**Published:** 2019-09-16

**Authors:** Fuming Zhang, Langhong Zheng, Shuihong Cheng, Yanfei Peng, Li Fu, Xing Zhang, Robert J. Linhardt

**Affiliations:** 1 Department of Chemical and Biological Engineering, Center for Biotechnology and Interdisciplinary Studies, Rensselaer Polytechnic Institute, Troy, NY 12180, USA; 2 Department of Chemistry and Chemical Biology, Center for Biotechnology and Interdisciplinary Studies, Rensselaer Polytechnic Institute, Troy, NY 12180, USA; zhenglanhong@126.com (L.Z.); csh_04@163.com (S.C.); yanfeipeng@ouc.edu.cn (Y.P.); ful3@rpi.edu (L.F.); xingzhang-cas@hotmail.com (X.Z.); 3 School of Pharmacy, Shanghai University of Medicine & Health Sciences, Shanghai 201318, China; 4 CAS Key Laboratory of Pathogenic Microbiology and Immunology, Institute of Microbiology, Chinese Academy of Sciences, Beijing 100101, China; 5 College of Marine Life Sciences, Ocean University of China, 5 Yushan Road, Qingdao 266003, China; 6 School of Food Science and Pharmaceutical Engineering, Nanjing Normal University, Wenyuan Road 1, Nanjing 210023, China; 7 Departments of Biology and Biomedical Engineering, Center for Biotechnology and Interdisciplinary Studies, Rensselaer Polytechnic Institute, Troy, NY 12180, USA

**Keywords:** growth factor, glycosaminoglycans, heparin, interaction, surface plasmon resonance

## Abstract

Most growth factors are naturally occurring proteins, which are signaling molecules implicated in cellular multiple functions such as proliferation, migration and differentiation under patho/physiological conditions by interacting with cell surface receptors and other ligands in the extracellular microenvironment. Many of the growth factors are heparin-binding proteins (HBPs) that have a high affinity for cell surface heparan sulfate proteoglycans (HSPG). In the present study, we report the binding kinetics and affinity of heparin interacting with different growth factors, including fibroblast growth factor (FGF) 2,7,10, hepatocyte growth factor (HGF) and transforming growth factor (TGF β-1), using a heparin chip. Surface plasmon resonance studies revealed that all the tested growth factors bind to heparin with high affinity (with K_D_ ranging from ~0.1 to 59 nM) and all the interactions are oligosaccharide size dependent except those involving TGF β-1. These heparin-binding growth factors also interact with other glycosaminoglycans (GAGs), as well as various chemically modified heparins. Other GAGs, including heparan sulfate, chondroitin sulfates A, B, C, D, E and keratan sulfate, showed different inhibition activities for the growth factor-heparin interactions. FGF2, FGF7, FGF10 and HGF bind heparin but the 2-*O*-sulfo and 6-*O*-sulfo groups on heparin have less impact on these interactions than do the *N*-sulfo groups. All the three sulfo groups (*N*-, 2-*O* and 6-*O*) on heparin are important for TGFβ-1-heparin interaction.

## 1. Introduction

Growth factors are proteins with activities for stimulating cellular growth, proliferation and differentiation by conducting specific cellular responses in a biological environment [1]. They play crucial roles in regulating a variety of physiological processes such as apoptosis, immunological or hematopoietic response, morphogenesis, angiogenesis, metabolism and wound healing. The abnormal activities altered expression or imbalance of growth factors can cause various diseases such as cancer, liver and lung fibrosis, and asthma. [2,3,4,5]. Each growth factor exerts its biological functions through the binding to its specific receptor and then activating associated downstream signaling pathway [6]. Based on the structural and functional characteristics on regulating specific types of cells and tissues, growth factors can be grouped into many different superfamilies/families such as: transforming growth factor β (TGF-β) superfamily, fibroblast growth factor (FGF) family, platelet-derived growth factor (PDGF) family, vascular endothelial growth factor (VEGF) family, epidermal growth factor (EGF) family, hepatocyte growth factor (HGF) family and neurotrophins family. Growth factors have been increasingly used in the treatment of many diseases, such as hematologic and oncologic diseases, cardiovascular diseases and tissue engineering [7,8,9,10,11].

**Figure 1:**
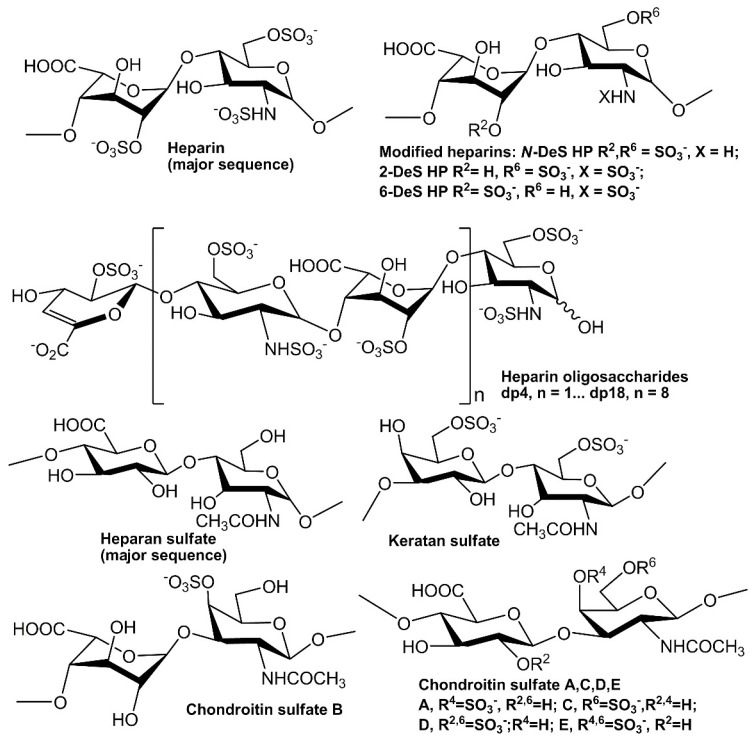
Chemical structures of heparin and heparin-derived oligosaccharides and GAGs.

In extracellular matrix, glycosaminoglycans (GAGs) are a family of linear polysaccharides that are typically highly sulfated and typically found as proteoglycans attached to membrane-associated core proteins [12]. Based on their disaccharide compositions (Figure 1), GAGs are classified into four groups: heparin/heparan sulfate (HS), chondroitin sulfate/dermatan sulfate, keratan sulfate and hyaluronic acid [13]. Heparin, HS and other GAGs have been reported to interact with various proteins giving profound effects on numerous physiological and pathophysiological processes including blood coagulation, cell growth and differentiation, host defense, lipid transport and metabolism, cell-to-cell and cell-to-matrix signaling, inflammation and cancer [14,15,16,17,18,19,20]. Many growth factors/receptors have been reported to interact with heparin/HS and other GAGs [21]. For example, more than 22 members of FGFs have been identified as heparin-binding proteins (HBPs). The interaction between FGF2 and heparin/HS is one of the best-studied examples of protein–GAG interaction. The well-characterized FGF2-heparin interaction shows an affinity (K_D_) at the nM level and the minimal binding size in heparin for FGF2 corresponds to a pentasaccharide with the *N*-sulfo and 2-*O*-sulfo groups critically important for the FGF2 binding. Two ternary, but different crystal structures of the 2:2:2 FGF2:FGF receptor (R) 1:heparin complex and 2:2:1 complex have been reported [22,23]. 

In the current study, we utilize surface plasmon resonance (SPR) to measure the binding kinetics and affinities of five different kind of growth factors, FGF2, FGF7, FGF10, HGF and TGF β1 with heparin using a heparin SPR chip. Competitive SPR studies using different chain lengths of heparin-derived oligosaccharides and different chemically modified heparins were conducted to determine the chain-size dependence and the effect of heparin sulfo group substitution on growth factor-heparin interactions. In addition, competition studies between the heparin on the chip surface and other GAGs in the solution were performed to determine the binding preferences of growth factors to different GAGs. A more comprehensive understanding of growth factor and heparin/GAG interactions at the molecular level is fundamentally important to understand the biological roles of these two important groups of biomolecules.

## 2. Results

### 2.1. Kinetics Measurements of Growth Factor–Heparin Interactions

**Figure 2:**
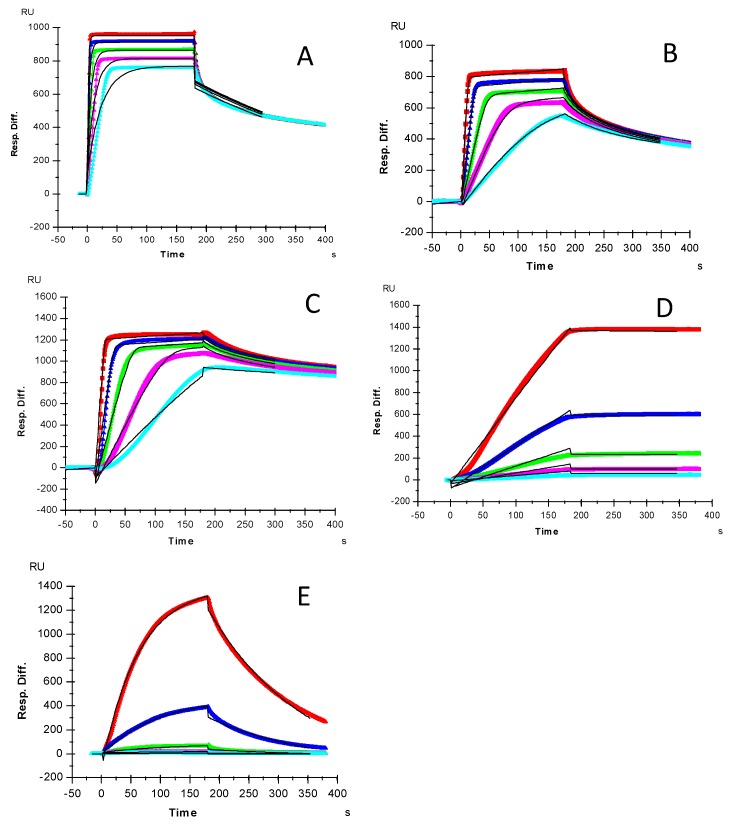
SPR sensorgrams for binding kinetics/affinity measurements for growth factor-heparin interactions: (**A**) FGF2, concentrations of the FGF2 (from top to bottom) were 32, 16, 8, 4, and 2 μM, respectively; (**B**) FGF7, concentrations of the FGF7 (from top to bottom) were: 250, 125, 63, 32 and 16 nM, respectively; (**C**) FGF10, concentrations of the FGF10 (from top to bottom) were: 250, 125, 63, 32 and 16 nM, respectively; (**D**) HGF, concentrations of HGF (from top to bottom): 20, 10, 5, 2.5 and 1.25 nM, respectively. (**E**) TGFβ-1, concentrations of TGFβ-1 (from top to bottom): 100, 50, 25, 12.5 and 6.3 nM, respectively. The black curves are the fitting curves using a 1:1 Langmuir model from BIAevaluation 4.0.1.

Kinetics measurements of growth factor–heparin interactions were carried out using a sensor chip with immobilized heparin. Sensorgrams of growth factor–heparin interaction are shown in Figure 2. The sensorgrams were fit globally to obtain apparent on (*ka*) and off (*kb*) rates for the binding equilibrium (Table 1), using the BIAEvaluation software and assuming a 1:1 Langmuir model. Among the 5 growth factors, due its extremely slow disassociate rate, HGF gives the highest affinity to heparin (K_D_ = 0.12 nM). The shapes of sensorgrams (Figure 2A–C) of FGF2, FGF7 and FGF10-heparin interactions seem to be similar and to have a comparable nM K_D_. 

### 2.2. Solution Competition Study on the Interaction between Heparin (Surface) and Growth Factor to Heparin-Derived Oligosaccharides (in Solution) Using SPR

Solution/surface competition experiments were performed by SPR to examine the effect of saccharide chain size of heparin on the growth factor–heparin interaction. Different size heparin-derived oligosaccharides (from dp4 to dp18) were used in the competition study. The same concentration (1000 nM) of heparin oligosaccharides were present in the growth factor–heparin interaction solution. 

**Figure 3:**
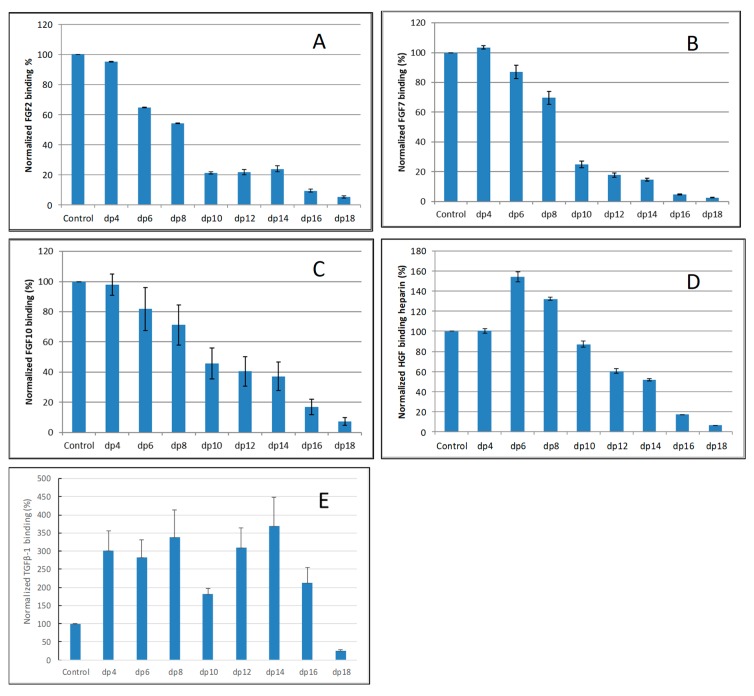
Bar graphs of normalized different growth factors binding preference to surface heparin by competing with different size of heparin oligosaccharides (from fp4 to dp18). (**A**) FGF2, (**B**) FGF7, (**C**) FGF10, (**D**) HGF, (**E**) TGFβ-1. Concentrations were 200, 100, 100, 20, and 50 nM for FGF2, FGF7, FGF10, HGF and TGFβ-1, respectively, and concentrations of heparin oligosaccharides were 1000 nM. All bar graphs based on triplicate experiments.

For FGF2-heparin interaction, weak competition effect was observed when 1000 nM of heparin tetrasaccharides (dp 4) present in the solution (Figure 3A). When the size of the oligosaccharide increased to dp6, the binding of FGF2 to the surface heparin obviously decreased. The variation of the FGF2 binding observed under competition suggests that the interactions between the FGF2 and heparin are chain-length dependent and the minimum binding size for the interaction is dp4.

For both FGF7 and FGF10, no competition effect was observed when 1000 nM of heparin tetrasaccharides dp4 was present in the solution (Figure 3B,C). When the size of the oligosaccharide was increased to dp6, the binding of FGF7 or FGF10 to the surface heparin obviously decreased. The interactions between the FGF7/FGF10 and heparin are chain-length dependent, and the minimum binding size for the interaction is dp6.

For HGF–heparin interaction, no competition effect was detected when 1000 nM of heparin dp4 to dp8 present in the solution (Figure 3D). When the size of the oligosaccharide was increased to dp10, the binding of HGF to the surface heparin obviously decreased. The variation of the HGF binding under competition with different sizes of heparin oligosaccharide suggests that the interactions between the HGF and heparin are chain-length dependent, and the minimum binding size for the interaction is dp10.

For TGFβ-1–heparin interaction, no competition effect pattern was detected when 1000 nM of heparin dp4 to dp 16 was present in the solution (Figure 3E)—it was surprising to note that the binding signals increased when these heparin oligosaccharides were present in the TGFβ-1 solutions. Only when the size of the oligosaccharide was increased to dp18 did an obviously decreased binding of TGFβ-1 to the surface heparin occur. 

### 2.3. SPR Solution Competition Study of Different Chemically Modified Heparins 

**Figure 4:**
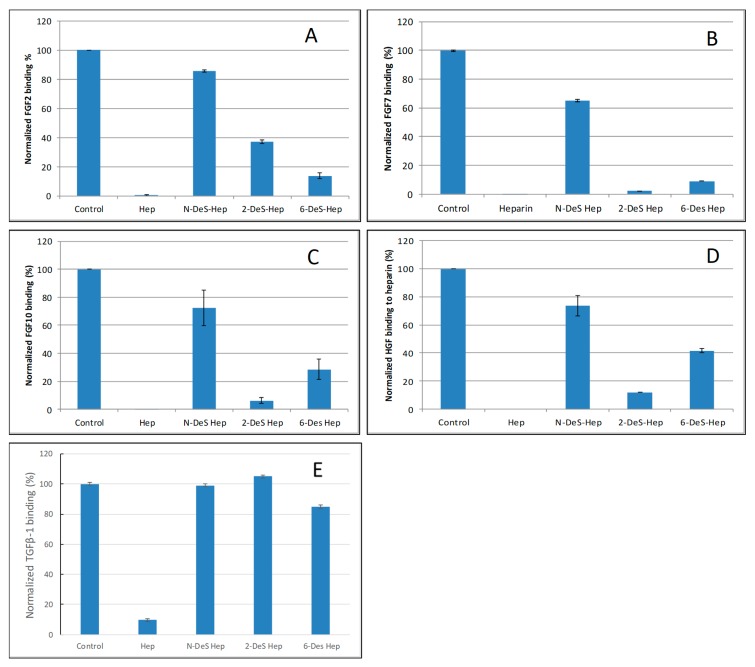
Bar graphs of normalized different growth factors binding preference to surface heparin by competing with different chemical modified heparin in solution. (**A**) FGF2, (**B**) FGF7, (**C**) FGF10, (**D**) HGF, (**E**) TGFβ-1. Concentrations were 200, 100, 100, 20, and 50 nM for FGF2, FGF7, FGF10, HGF and TGFβ-1, respectively, and concentrations of different modified heparin were 1000 nM. All bar graphs based on triplicate experiments.

SPR competition bar graphs of the chemical modified heparin competition levels are displayed in Figure 4. For FGF2, FGF7, FGF10 and HGF, all the three chemical modified heparins (*N*-desulfated heparin and 2-*O*-desulfated heparin and 6-*O*-desulfated heparin) showed reduced inhibitory activities (Figure 4A–D). Much higher reduced inhibitory activities were observed for *N*-desulfated heparin than 2-*O*-desulfated heparin and 6-*O*-desulfated heparin suggesting 2-*O*-sulfo and 6-*O-*sulfo groups on heparin have less impact on these interactions. For TGFβ-1, all the three chemical modified heparins greatly lost the inhibitory activities suggesting *N*-sulfo, 2-*O*-sulfo and 6-*O-*sulfo groups on heparin are important for TGFβ-1-heparin interaction (Figure 4E).

### 2.4. SPR Solution Competition Study of Different GAGs

**Figure 5:**
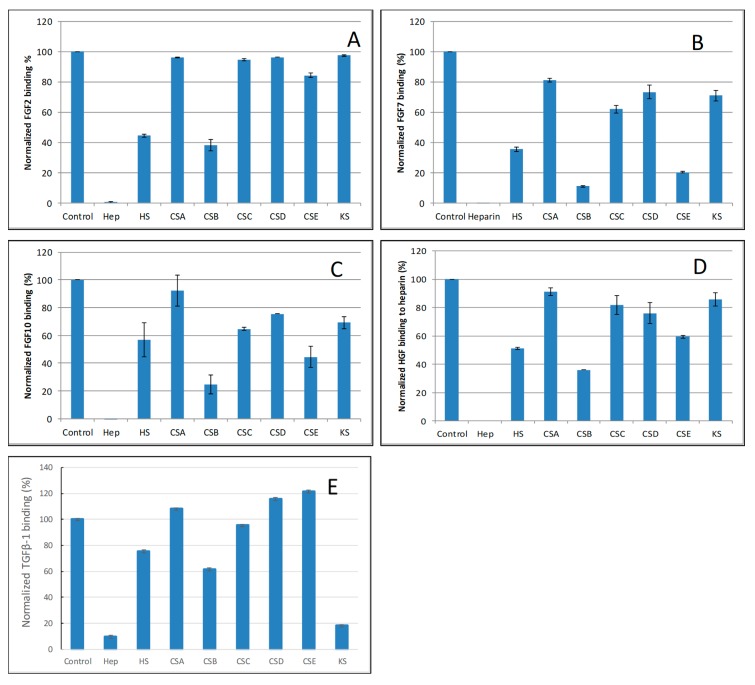
Bar graphs of normalized different growth factors binding preference to surface heparin by competing with different GAGs in solution. (**A**) FGF2, (**B**) FGF7, (**C**) FGF10, (**D**) HGF, (**E**) TGFβ-1. Concentrations were 200, 100, 100, 20, and 50 nM for FGF2, FGF7, FGF10, HGF and TGFβ-1, respectively and concentrations of GAGs were 1000 nM. All bar graphs based on triplicate experiments.

The SPR competition assay was also utilized to determine the binding preference of growth factors to various GAGs (Figure 1). SPR competition bar graphs of the GAG competition levels are displayed in Figure 5. 

For FGF2, heparin produced the strongest inhibition by competing >98% of FGF2 binding to immobilized heparin. Strong inhibitory activities (>60%) were observed for HS and CSB. Weak inhibitory activities for CSA, CSC, CSD, CSE and KS were observed (Figure 5A).

For FGF7, heparin produced the strongest inhibition by competing >95% of FGF7 binding to immobilized heparin. Very strong inhibitory activities (>80%) were observed for CSB, and CSE. Modest inhibitory activities (>40%) for HS, weak inhibitory activities for CSA, CSC, CSD and KS were observed (Figure 5B).

For FGF10, heparin produced the strongest inhibition by competing 100% of FGF10 binding to immobilized heparin. Very strong inhibitory activities (~80%) were observed for CSB, strong inhibitory activities for HS, and CSE, weak inhibitory activities for CSC, CSD and KS, no inhibitory activity for CSA was observed (Figure 5C).

For HGF, heparin produced the strongest inhibition by competing with 100% of HGF binding to immobilized heparin. Strong inhibitory activities (>60%) were observed for HS and CSB. Modest inhibitory activity (>40%) was observed for CSE. Weak inhibitory activities for CSA, CSC, CSD, and KS were observed (Figure 5D).

For TGFβ-1, heparin produced the strongest inhibition by competing >90% of TGFβ-1 binding to immobilized heparin. Very strong inhibitory activity (>80%) was observed for KS. While modest inhibitory activities for HS and CSB, no inhibitory activity for CSA CSC, CSD and CSE were observed (Figure 5E).

## 3. Discussion

The biological functions of ECM are largely facilitated by the networks of protein–protein and protein–GAG interactions [24]. There have been intensive biochemical, cell biological and genetic studies have shown GAG to play crucial roles in regulating most growth factor-related signaling pathways, such as the pathways for transforming growth factor-β (TGF-β), and fibroblast growth factors (FGFs) [25,26,27,28,29]. While these studies have provided some level of understanding of growth factor–GAG interactions, there is still a lack of detailed structural and biophysical data about these interactions. A more comprehensive understanding of growth factor and GAG interactions at the molecular level is vitally important to translate these drugable proteins from biologically active in vitro to potential medicines. 

To explore the GAG–protein interactome, the characteristic features include kinetics (association and dissociation rates) and affinity (K_D_), thermodynamic parameters, GAG-binding sequences, protein-binding motifs, chain size, sulfation pattern and epimerization. Many techniques have been used to study GAG–protein interactions for both qualitative and quantitative, structural and kinetic information. These techniques include: SPR, NMR spectroscopy, isothermal titration calorimetry (ITC), filter-binding assays, affinity chromatography, X-ray crystallography, fluorescence spectroscopy, electrophoresis, molecular modeling and glycan microarray [15,16,17,18]. SPR is a powerful biosensor approach for studying GAG–protein interactions in real-time and a label-free environment. 

Many previous reports have shown that all five of the selected growth factors (FGF2, FGF7, FGF10, HGF and TGF β-1) in this study are heparin-binding proteins [22,23,25,26,27,28,29,30,31,32,33,34,35,36,37,38]. Most of those studies only provided qualitative information regarding these interactions. In this study, we utilized the SPR system to measure the binding kinetics/affinity and structural characteristic of five selected growth factor-heparin/GAG interactions. SPR analysis demonstrated that all of these growth factors bind to heparin with high affinity (with K_D_ ranging from ~0.1 to 59 nM) (Figure 2 and Table 1). Our kinetics and affinity data agree with most of the previous reports [26,27,28,29]. Asada et al. [26] assessed the affinities of all known FGFs (FGF1–FGF23) to heparin using heparin-Sepharose columns. They found that all FGF members except the FGF19 subfamily (FGF15, 19, 21 and 23) had strong affinities with heparin, as indicated by the requirement of 1.0–1.5 M NaCl for the elution from heparin-Sepharose columns. Among the five tested growth factors, HGF showed the highest affinity to heparin (K_D_ = 0.12 nM), which is comparable to the lower side of the previous report by Rahmoune, et al. [28]. Based on IAsys resonant mirror biosensor assay, they obtained the apparent affinity ranging from 0.2 nM to 2.8 nM for the HGF binding to immobilized different HS purified from mammary cells. It seems that the high affinity for HGF–heparin interaction measured in our study is due to the extremely slow disassociate rate (k_d_ = 5.7 × 10^−7^ L/s). In comparison with FGF2, 7 and 10, TGFβ-1 is a weaker heparin binding protein with affinity (K_D_ = 59 nM), which is in agreement with the reported results using heparin-Sepharose column showing that the TGFβ-1 was eluted with a lower concentration of NaCl (0.9–1.2 M) [29]. Since SPR has limitations with respect to determining the stoichiometry of molecular interactions, we only used a simple 1:1 Langmuir model to process the binding data of these growth factor-heparin interactions. 

To test the structure preference of growth factor–GAG interactions, SPR competition experiments were performed to see the impact of heparin structure (e.g., chain size, sulfo group position) on the interactions (See Table 2 and Figure 3 and Figure 4). The competition SPR studies with different-sized heparin oligosaccharides revealed that the FGF2, 7, 10 and HGF–heparin interactions were chain-length dependent, and demonstrated a minimum heparin oligosaccharide length greater than dp4, dp6 and dp 10, respectively. These results are in agreement with most of the previous reports [27,33,34,35]. For example, it was reported that heparin/HS dp4–dp10 or dp4–dp8 is the minimal chain size required for interaction with FGF2 or FGF7/FGF10 [28]. For the TGFβ-1–heparin interaction, the results showed less/no chain size dependence when the heparin size was <dp 16, and large-sized heparin (≥dp18) was required to bind TGFβ-1, which is different from the other growth factors tested in this study. 

The SPR competition experiments with chemically modified heparin clearly showed growth factor–heparin interaction is impacted by the sulfation position (see Table 2 and Figure 5). For FGF2, FGF7, FGF10 and HGF binding to heparin, 2-*O*-sulfo and 6-*O*-sulfo groups on heparin have less impact on these interactions than do the *N*-sulfo groups. FGF2 and FGF7 showed a similar sulfation preference pattern: NS > 2S > 6S; whereas FGF10 and HGF showed NS > 6S > 2S. All three sulfo groups (*N*-, 2-*O* and 6-*O*) on heparin are important for the TGFβ-1–heparin interaction (NS ≈ S2S ≈ 6S). Our results partially agree with those reported in [25,30]. Using the octasaccharides library, Ashikari-Hada et al. reported that FGF-2 needed 2S by not 6S; FGF-7 required both 2S and 6S; FGF-10 needed 6S but not 2S; HGF needed both 2S and 6S. Competition assays in SPR biosensor demonstrated that TGF-β1 required NS and 6S for binding [36]. The difference might be due to the different source of chemically modified heparin used.

The SPR competition assay using various other GAGs revealed the binding preference of growth factors. For FGF2, FGF7, FGF10 and HGF, they showed a similar GAG binding preference pattern: Hep >> HS ≈ CSB >> CSE > CSA, C, D, KS. These results are in partial agreement with the reported preference of FGFs for GAGs showing HS and CSB (DS) binding FGF2, 7 and 10 [26]. It was reported that HGF has a cofactor requirement for binding HS and DS in the activation of its signaling receptor MET [38]. It should be noted that KS is reported for the first time to show a strong binding to TGF-β1.

## 4. Materials and Methods

### 4.1. Materials

Recombinant human FGF2 was a gift from Amegen; FGF7 and FGF10 were generously provided by Professor Mohammadi from NYU; HGF and TGF β-1 were purchased from R&D Systems (Minneapolis, MN, USA). The GAGs used were porcine intestinal heparin and porcine intestinal heparan sulfate from Celsus Laboratories (Cincinnati, OH, USA); chondroitin sulfate A from porcine rib cartilage (Sigma, St. Louis, MO, USA); chondroitin sulfate B from porcine intestine (Sigma), chondroitin sulfate C from shark cartilage (Sigma); chondroitin sulfate D from whale cartilage (Seikagaku, Tokyo, Japan); chondroitin sulfate E from squid cartilage (Seikagaku); and keratan sulfate which was isolated from bovine cornea in our lab. *N*-desulfated heparin (*N*-DeS HP) and 2-*O*-desulfated heparin (2-DeS HP) were all prepared based on Yates et al. [39]. The 6-*O*-desulfated heparin (6-DeS HP) was provided by Professor Lianchun Wang from USF. Heparin oligosaccharides included tetrasaccharide (dp4), hexasaccharide (dp6), octasaccharide (dp8), decasaccharide (dp10), dodecasaccharide (dp12), tetradecasaccharide (dp14), hexadecasaccharide (dp16) and octadecasaccharide (dp18) and were prepared from porcine intestinal heparin controlled partial heparin lyase 1 treatment of followed by size fractionation [40]. The chemical structures of these GAGs are shown in Figure 1. Sensor SA chips were from BIAcore (GE Healthcare, Uppsala, Sweden). SPR measurements were performed on a BIAcore 3000 operated using BIAcore 3000 control and BIAevaluation software (version 4.0.1, GE Healthcare, Uppsala, Sweden). 

### 4.2. Preparation of Heparin Biochip 

The preparation of biotinylated heparin was followed our previous protocol with minor modification [41]: in 200 µL of H_2_O, 2 mg of heparin and 2 mg of amine–PEG3–Biotin (Thermo Scientific, Waltham, MA, USA) were mixed with 10 mg of NaCNBH_3_. The initial reaction was carried out at 70 °C for 24 h, and then a further 10 mg of NaCNBH_3_ was added to continue running the reaction for another 24 h. After completing the reaction, the mixture was desalted with a spin column (3000 molecular weight cut-off). Biotinylated heparin was freeze-dried for chip preparation. The biotinylated heparin was immobilized to a streptavidin (SA) chip based on the manufacturer’s protocol. In brief, a 20 μL solution of the heparin-biotin conjugate (0.1 mg/mL) in HBS-EP buffer (0.01 M HEPES, 0.15 M NaCl, 3 mM EDTA, 0.005% surfactant P20, pH 7.4) was injected over flow cell 2 (FC2), 3 (FC3) and 4 (FC4) of the SA chip at a flow rate of 10 μL/min. The successful immobilization of heparin was confirmed by the observation of a ~100 resonance unit (RU) increase in the sensor chip. The control flow cell (FC1) was prepared by 1 min injection with saturated biotin.

### 4.3. Kinetic Measurement of Interaction between Growth Factor and Heparin Using BIAcore 

FGF2, FGF7, FGF10, HGF and TGF β-1, samples were diluted in HBS-EP buffer (0.01 M HEPES, 0.15 M NaCl, 3 mM EDTA, 0.005% surfactant P20, pH 7.4). Different dilutions of growth factors were injected at a flow rate of 30 µL/min. At the end of the sample injection, the same buffer was flowed over the sensor surface to facilitate dissociation. After a 3 min dissociation time, the sensor surface was regenerated by injecting with 30 µL of 0.25% SDS or 2 M NaCl to fully regenerate the surface. The response was monitored as a function of time (sensorgram) at 25 °C. 

### 4.4. Solution Competition Study between Heparin on the Chip Surface and Heparin-Derived Oligosaccharides in Solution Using SPR

Growth factors (in concentrations ranging from 20 nM to 200 nM) mixed with 1000 nM of heparin oligosaccharides, including tetrasaccharide (dp4), hexasaccharide (dp6), octasaccharide (dp8), decasaccharide (dp10), dodecasaccharide (dp12), tetradecasaccharide (dp14), hexadecasaccharide (dp16) and octadecasaccharide (dp18) in HBS-EP buffer, were injected over the heparin chip, each at a flow rate of 30 μL/min. After each run, the dissociation and the regeneration steps were performed as described above. For each set of competition experiments on SPR, a control experiment (only protein without any heparin or oligosaccharides) was performed to make sure the surface was completely regenerated, and that the results obtained between runs were comparable. 

### 4.5. Solution Competition Study between Heparin on Chip Surface and GAGs, Chemical Modified Heparin in Solution Using SPR

For the testing of inhibition of other GAGs and chemically modified heparins to the growth factor–heparin interaction, growth factors were pre-mixed with 1000 nM of GAG or chemically modified heparin and injected over the heparin chip at a flow-rate of 30 μL/min. After each run, a dissociation period and regeneration protocol were performed as described above. 

## 5. Conclusions

SPR studies revealed that all five tested bind to heparin with high affinity (with K_D_ ranging from ~0.1 to 59 nM) and all of the interactions are oligosaccharide size dependent except TGF β-1. FGF2, FGF7, FGF10 and HGF bind heparin with *N*-sulfo groups. All the three sulfo groups (*N*-, 2-*O* and 6-*O*) on heparin are important for TGFβ-1–heparin interaction. Other GAGs, including HS, CS A, B, C, D, E and KS, showed different inhibition activities for the growth factor–heparin interactions. We believe this study on characteristic features of growth factor–GAG interactome could be key determinants of their specific biological activities.

## Figures and Tables

**Table 1:** Summary of kinetic data of growth factor-heparin interactions *.

Interactions	k_a_ *(1/MS)*	k_d_ *(1/S)*	*K_D_ (M)*
FGF2/Heparin	2.4 × 10^6^ (±5.9 × 10^4^)	2.9 × 10^−3^ (±4.7 × 10^−5^)	1.2 × 10^−9^
FGF7/Heparin	5.2 × 10^6^ (±7.2 × 10^4^)	0.025 (±3.4 × 10^−4^)	4.9 × 10^−9^
FGF10/Heparin	5.7 × 10^6^ (±1.7 × 10^4^)	7.1 × 10^−3^ (±2.1 × 10^−5^)	1.3 × 10^−9^
HGF/Heparin	4.2 × 10^3^ (±104)	5.7 ×10^−7^ (±3.6 × 10^−6^)	1.4 × 10^−10^
TGFβ-1/heparin	1.0 × 10^5^ (±342)	9.2 × 10^−3^ (±4.0 × 10^−5^)	5.9 × 10^−8^

* The data with (±) in parentheses are the standard deviations (SD) from global fitting of five injections using a 1:1 Langmuir model.

**Table 2:** Summary of structure preference of growth factor–GAG interactions.

Interactions	Size	Sulfation	*GAGs*
FGF2/Heparin	>dp4	NS > 2S > 6S	Hep >> HS ≈ CSB >> CSE > CSA, C, D, KS
FGF7/Heparin	>dp6	NS > 2S ≈ 6S	Hep >> CSB > HS > CSE > CSA, C, D, KS
FGF10/Heparin	>dp6	NS > 6S > 2S	Hep >> CSB > HS > CSE > CSA, C, D, KS
HGF/Heparin	>dp10	NS > 6S > 2S	Hep >> CSB > HS > CSE > CSA, C, D, KS
TGFβ-1/heparin	>dp18	NS ≈ 2S > 6S	Hep > KS > CSB > HS > CSA, C, D, E

## Abbreviations

GAG	glycosaminoglycan
SPR	surface plasmon resonance
HS	heparan sulfate
CSA	chondroitin sulfate A
CSB	chondroitin sulfate B
CSC	chondroitin sulfate C
CSD	chondroitin sulfate D
CSE	chondroitin sulfate E
KS	keratan sulfate
SA	streptavidin
dp	degree of polymerization
FGF	fibroblast growth factors
HGF	hepatocyte growth factor
TGF	transforming growth factor
